# Butenolide, a Marine-Derived Broad-Spectrum Antibiofilm Agent Against Both Gram-Positive and Gram-Negative Pathogenic Bacteria

**DOI:** 10.1007/s10126-018-9861-1

**Published:** 2019-01-05

**Authors:** Qi Yin, Jinyou Liang, Weipeng Zhang, Lv Zhang, Zhang-Li Hu, Yu Zhang, Ying Xu

**Affiliations:** 1 Shenzhen Key Laboratory of Marine Bioresource and Eco-environmental Science, Shenzhen Engineering Laboratory for Marine Algal Biotechnology, College of Life Sciences and Oceanography, Shenzhen University, 3688 Nanhai Avenue, Nanshan Section, Shenzhen, 518060 People’s Republic of China; 2 State Key Laboratory of Biotherapy/Collaborative Innovation Center for Biotherapy, West China Hospital, West China Medical School, Sichuan University, No. 17, Section 3, South Renmin Road, Chengdu, Sichuan 610041 People’s Republic of China; 3 Division of Life Science, Hong Kong University of Science and Technology, Hong Kong, SAR People’s Republic of China

**Keywords:** Butenolide, Antibiofilm agent, Antibiotics enhancer, Drug discovery

## Abstract

Bacterial biofilm can cause nosocomial recurrent infections and implanted device secondary infections in patients and strongly promotes development of pathogenic drug resistance in clinical treatments. Butenolide is an effective anti-macrofouling compound derived from a marine *Streptomyces* sp., but its antibiofilm efficacy remains largely unexplored. In the present study, the antibiofilm activities of butenolide were examined using biofilms formed by both Gram-positive and Gram-negative pathogenic model species. Four *Escherichia coli* strains, *Pseudomonas aeruginosa*, and methicillin-resistant *Staphylococcus aureus* (MRSA) were used as targets in antibiofilm assays that examined the effects of butenolide, including the following: (i) on bacterial growth; (ii) in inhibiting biofilm formation and eradicating mature biofilm; (iii) on biofilm structures. In addition, the synergistic effect between butenolide with tetracycline was also examined. Butenolide not only effectively inhibited the biofilm formation but also eradicated pre-formed biofilms of tested bacteria. Fractional inhibitory concentration index (FICI) indicated that butenolide was a potential tetracycline enhancer against *E. coli*, *P. aeruginosa*, and MRSA. These results indicated that butenolide may hold a great potential as an effective antibiofilm agent to control and prevent biofilm-associated infections in future clinical treatments.

**Electronic supplementary material:**

The online version of this article (10.1007/s10126-018-9861-1) contains supplementary material, which is available to authorized users.

## Introduction

Bacterial biofilms are a highly structured consortium surrounded by self-secreted extracellular polymeric substances (EPSs, mainly include polysaccharides, proteins, DNA, and lipids), which can attach to almost all surfaces. Rather than the planktonic state, the attached state is an important survival strategy for most microorganisms (Costerton et al. 1999; Hall-Stoodley et al. 2004; Kolter 2010; O’Toole et al. 2000). Biofilms act as efficient protective barriers, hinder permeation of drugs, and cause fast translation and acquisition of drug resistance genes among bacterial populations. Consequently, bacteria in biofilm become more resistant to antibiotics (10–1000 times) and to host immune defense than those in planktonic state both in vitro and in vivo (Fux et al. 2005; Gilbert et al. 2002; Høiby et al. 2010; Høiby et al. 2011).

It is estimated that around 80% of microbial infections were associated with biofilm formation (National Institutes of Health 2002), including surface infections of wounds and organs, and serious implanted device-derived secondary infections (Bryers 2008; Francolini and Donelli 2010; Wu et al. 2015a). Though biofilm infections are highly recalcitrant to bactericidal agents, planktonic cells derived from biofilm are fully susceptible to antibiotics in most cases (Lewis 2006; Lewis 2010). As such, antibiofilm agents, if applied as enhancers with antibiotics, may be a promising solution to treat biofilm-related infections (de la Fuente-Nunez et al. 2013; Rabin et al. 2015; Ramritu et al. 2008; Wu et al. 2015a).

So far, the most well-studied natural antibiofilm agents are the brominated furanones derived from the red alga *Delisea pulchra* (de Nys et al. 1993; Brackman and Coenye 2015). These small molecules and their synthetic derivatives exhibited potent inhibitory activity against biofilm formation of many hostile (opportunistic) pathogens, including *Escherichia coli* (Ren et al. 2001; Ren et al. 2004b), *Pseudomonas aeruginosa* (Hentzer et al. 2002; Hentzer et al. 2003), *Streptococci* spp. (Lönn-Stensrud et al. 2007), *Staphylococcus epidermidis* (Hume et al. 2004; Lönn-Stensrud et al. 2008), and *Salmonella enterica* (Janssens et al. 2008). Studies have shown that these furanone compounds could reduce biofilm formation through inhibition of Quorum Sensing (QS), bacterial cell-to-cell communication systems mediated by AI-2 and AHLs in Gram-negative and/or Gram-positive bacteria, respectively (Lasarre and Federle 2013; Kuehl et al. 2009; Manefield et al. 2002; Ren et al. 2004a; Waters and Bassler 2005). However, the application of brominated furanones in clinics is still not practical due to several reasons, including ambiguous antibiofilm activities tests and toxicity to mammalian cells (Rabin et al. 2015). As such, there is an urgent need of novel, non-toxic, and broad-spectrum antibiofilm agents.

Butenolide (5-octylfuran-2(5H)-one, abbreviated as BU), structurally similar to the brominated furanones, has a core structure of 2-furanone ring, and a straight alkyl side-chain but no halogen. It was previously discovered as a non-toxic, effective antifouling compound derived from a marine *Streptomyces* sp. (Xu et al. 2010; Zhang et al. 2012). The antifouling activity relies not only on the 2-furanone ring but also the high lipophilicity from the straight alkyl side-chain (Xu et al. 2010). Although BU has been proven to be capable of inhibiting mixed species of biofilms in the natural marine environment in a recent study (Ding et al. 2018), the effect of BU on single pathogenic biofilm has not been examined. The core structure of its antibiofilm activity has not been determined either. In the present study, the effects of BU in inhibition and eradication of biofilm formation of several important pathogens including *E. coli*, *P. aeruginosa*, and methicillin-resistant *Staphylococcus aureus* (MRSA) were investigated*.* The synergistic antibiofilm effect between BU with tetracycline was also examined. Furthermore, the antibiofilm activities of two hydrophilic analogs of BU were also tested to confirm whether the lipophilicity of BU was critical to its antibiofilm activity.

## Materials and Methods

### Strains and Chemicals

Four *E. coli* strains, including the wide type strain K-12 ATCC 25404, quality control strain ATCC 25922, enterohemorrhagic strain O157:H7, and laboratory cloning strain DH5α (Takara, Japan), *P*. *aeruginosa* strain PAO1, and MRSA strain ATCC 43300 were used as biofilm model organisms. Chemicals and media were purchased from Sigma-Aldrich (Poole, UK), unless otherwise stated.

**Fig. 1:**
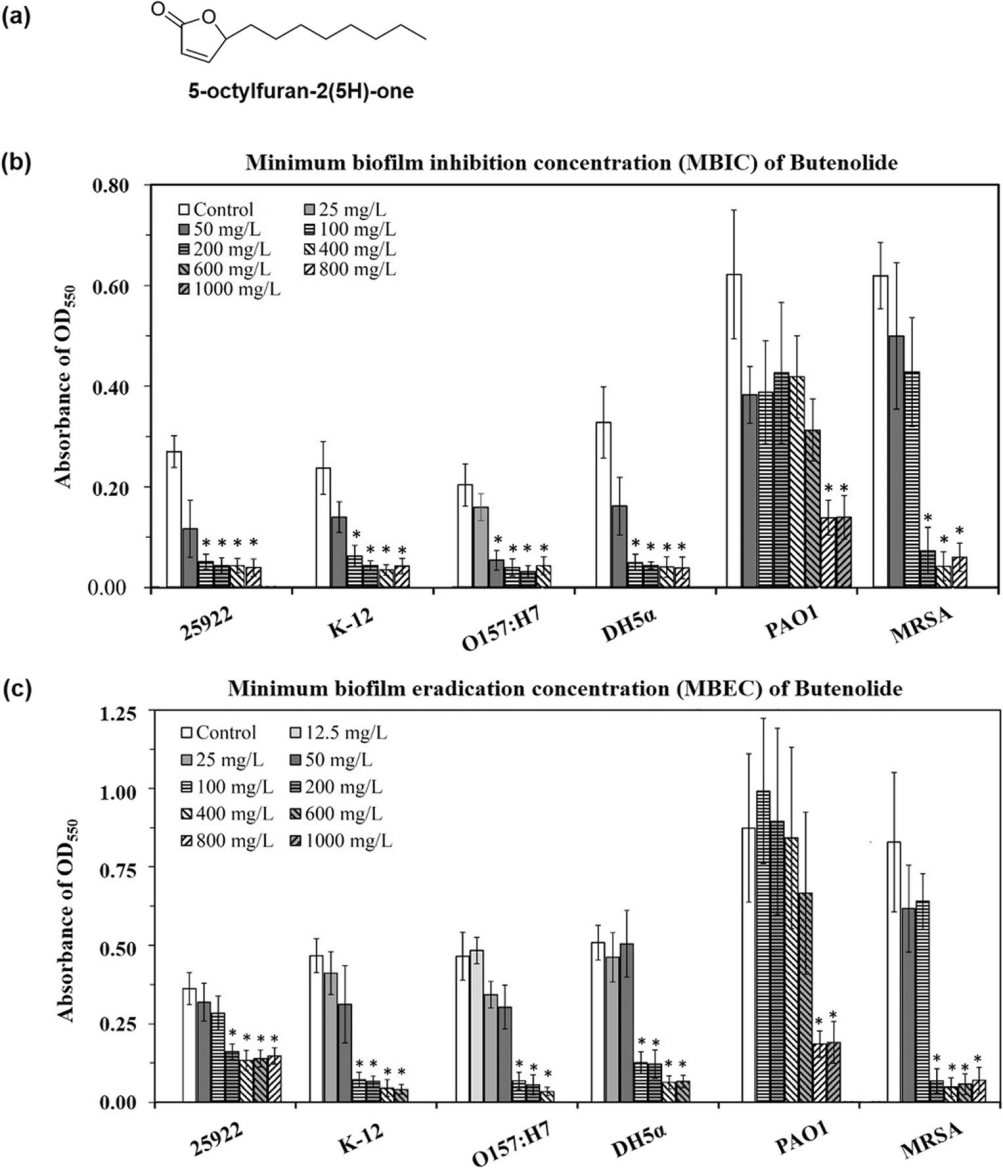
Bacterial viability in remained biofilm treated with butenolide (BU) was quantified by MTT assay. **a** Chemical structure of BU. **b** Bacteria were exposed to various concentrations of BU and incubated 24 h at 37 °C to detect its inhibitory efficiency of biofilm formation (MBIC assay). **c** After 24 h incubation at 37 °C to form mature biofilms, pre-formed biofilms were exposed to various concentrations of BU and incubated another 24 h at 37 °C, detecting its biofilm eradicating efficiency (MBEC assay). Asterisk represented significant (*p* < 0.01) differences compared to respective controls

Butenolide (abbreviated as BU) was synthesized by Shanghai Medicilon Inc. (Shanghai, China). The structure is shown in Fig. 1a.

### Antimicrobial Susceptibility Tests

The minimum inhibitory concentrations (MICs) of BU were determined according to the Clinical and Laboratory Standards Institute (CLSI) guideline M100-S25 (CLSI 2015). The individual MIC of each strain treated with various concentrations of BU was determined by OD_595_ using a spectrophotometer (Varioskan Flash, Thermo Scientific, USA). Briefly, 200 μL of bacterial culture using Lysogeny broth (LB) medium at a final concentration of 5 × 10^5^ CFU/mL was added into 96-well microplate (Corning, USA), followed by addition of 1 μL of BU dissolved in DMSO at final concentrations of 50, 100, 200, 500, 800, 1000, and 1200 mg/L. DMSO was used as the negative control. The growth-retarding concentration (GRC) was determined as a drug concentration that caused 30% decrease of OD_595_ of bacterial cells (Janssens et al. 2008). The GRC values of BU for each strain were determined at 16 h since BU affected bacterial growth most significantly at this time point for all strains except for PAO1, whose GRC value was determined at 12 h. All the experiments were performed in triplicates and repeated three times.

### Sole Carbon Source Tests

Bacterial cells were revived on LB agar plates, and the culture was grown overnight in LB at 37 °C. Overnight bacterial culture was diluted 1:500 (*v*/*v*) using M9 medium (Bren et al. 2016), then 200 μL of diluted culture was added into each well of the 96-well microplates. M9 containing 11 mM glucose and BU of equimolar carbon were used as the positive control and experimental group, respectively. The plates were then incubated at 37 °C for 36 h and OD_595_ was measured every 4 h. Each treatment had three triplicates and the experiment was repeated three times.

### Effect of BU on Biofilm Formation Inhibition

The minimum biofilm inhibitory concentration assay (MBIC assay) was performed by MTT staining assay. In this assay, bright yellow MTT [3-(4,5-dimethyl-2-thiazolyl)-2,5-diphenyl-2H-tetrazolium bromide; Himedia] could be reduced by activated succinate dehydrogenase in viable cell mitochondria to blueviolet formazan, which was read at 550 nm after being dissolved in DMSO. The intensity of the color was correlated to the number of viable cells in remaining biofilm (Nair et al. 2016). In brief, an overnight culture of each strain was diluted in LB broth with 0.5% glucose to achieve ~ 5 × 10^6^ CFU/mL. Diluted cultures with various concentrations of BU were added (1 mL/well) into a 24-well microplate (Corning, USA), incubated at 37 °C for 24 h. After incubation, the wells were rinsed twice by 1 × PBS to remove planktonic and non-adhering cells. MTT staining was conducted as previously described (Nair et al. 2016). The experiments were performed in triplicates and repeated three times. The MBIC was defined as the minimum concentration of BU that prevents biofilm formation, indicated by no color development.

### Effect of BU on Pre-formed Mature Biofilm Eradication

The bacterial biofilm eradication ability of BU (minimum biofilm eradication concentration assay, MBEC assay) was also conducted. After biofilm formation for 24 h following incubation steps in MBIC assay without addition of BU, each well was rinsed twice by 1 × PBS, challenged with a certain concentration of BU in 1 mL of LB broth with 0.5% glucose, and incubated for another 24 h at 37 °C. The viable cells in remaining biofilm were determined using the MTT assay. The experiments were performed in triplicates and repeated three times. The MBEC was defined as the minimum concentration of BU that eradicates pre-formed biofilms, indicated by no color development.

### CLSM and SEM Analysis of Biofilm Structure Under Treatment of BU

Bacterial biofilm was developed on glass cover slips placed in 24-well microplate for 24 h at 37 °C, under the MBIC of BU. After rinsed twice with 1 × PBS and air-dried for 30 min, the biofilms were stained using the BacLight Live/Dead Viability Kit (L7007, Invitrogen, USA) at 37 °C for 30 min in dark. The stained biofilms on glass slips were viewed using a laser scanning confocal microscopy (CLSM, LSM7 DUO 710+LIVE, Zeiss, Germany) at 488 and 561 nm. The COMSTAT analyses were used for quantitative analysis of the image stacks produced by CLSM (Heydorn et al. 2000). The evaluated parameters obtained for the live biofilm cells included biofilm average coverage, average thickness, and biovolume, whereas thresholding was automatically calculated as previously described (Castelo-Branco et al. 2016).

The effects of BU on biofilm matrix structure were analyzed using a scanning electronic microscope (SEM, SU-70, HITACHI, Japan). Strain *E. coli* K-12, PAO1, and MRSA ATCC 43300 were used as biofilm model species. Bacterial biofilms were developed on glass cover slips placed in a 24-well microplate for 24 h at 37 °C, under the MBICs of BU. Then, biofilms were dehydrated by a graded series of ethanol concentrations and subjected to SEM analysis.

### Quorum Sensing Inhibition Assays of BU

The AI-2 inhibition assays were conducted following the procedures as previously described (Taga and Xavier 2011). The reporter strain was *Vibrio harveyi* BB170, which could produce bioluminescence as QS product mediated by self-secreted AI-2 molecules. AHLs inhibition assays were conducted as previously described (Martinelli et al. 2004). *Chromobacterium violaceum* CV026 was used as a reporter bacterium for short-chain AHLs (C4–C8) (McClean et al. 1997), while *C. violaceum* VIR24 was used as a reporter bacterium for long-chain AHLs (C8–C14) (Someya et al. 2009). *C. violaceum* CV026 and VIR24 could produce violacein as QS product mediated by AHLs.

### Analysis of Synergistic Effect Between BU with Tetracycline

Possible synergistic/antagonistic effect of BU with tetracycline was assessed via a standard checkerboard assay (Odds 2003). In brief, MBECs of tetracycline against six tested strains were measured following MBEC assay. Different pairs of BU and tetracycline concentrations were combined to eradicate 24 h pre-formed biofilms and to determine the fractional inhibitory concentration index (FICI). The FICI of biofilm eradication was determined as following equation: (MBEC of drug A in combination/MBEC of drug A alone) + (MBEC of drug B in combination/MBEC of drug B alone). The effect was determined as synergistic when FICI ≤ 0.5, as additive when 0.5 < FICI ≤ 1.0, as indifferent when 1.0 < FICI ≤ 4.0, and as antagonistic when FICI > 4.0 (Odds 2003). The experiments were performed in triplicates and repeated three times.

### Effect of Lipophilicity to Antibiofilm Activity of BU

A conjugated exocyclic vinyl bromide and the furanone ring have been suggested to be critical to the antibiofilm activity of brominated furanones (Han et al. 2008). In contrast, BU lacks any halogen but has a highly lipophilic alkyl side-chain, which has been proved critical to its anti-macrofouling activity (Li et al. 2012; Xu et al. 2010). To confirm if the lipophilicity was also critical to the antibiofilm activity of BU, two hydrophilic analogs of BU, one with a carbonyl (5-(7-oxoctyl)furan-2(5H)-one, BUO) and the other with an hydroxyl (5-(7-hydroxyoctyl)furan-2(5H)-one, BUOH) on the seventh carbon in the alkyl side-chain of BU, respectively, were synthesized to test their antibiofilm activities against *E. coli* K-12 and MRSA ATCC 43300. The MBICs and MBECs of these two compounds against the target strains were measured following the steps described above.

### Statistical Analysis

Statistical comparisons were performed using one-way ANOVA followed by Turkey’s post-hoc analysis (SPSS software 16.0). Statistically significant results were depicted by *p* values less than 0.01.

## Results

### Bacteria Face No “Live or Dead” Selective Stress Under Treatment of BU

The antimicrobial ability of butenolide (abbreviated as BU) was assessed using MICs and sole carbon source tests. The results showed that BU has a low toxicity on bacterial growth of six tested strains because the GRCs of BU ranged from 500 to 1200 mg/L and all MICs were greater than 1200 mg/L (Table 1). All of the six tested strains could not use BU as sole carbon source (Fig. S2) in the sole carbon source tests.

**Table 1:** Antimicrobial activity of butenolide (BU) to planktonic state bacteria, antibiofilm efficiency of BU in inhibiting biofilm formation and eradicating 24 h pre-formed biofilm

Strain		GRC^a^ (mg/L)	MBIC_50_^b^ (mg/L)	MBIC (mg/L)	MBEC (mg/L)	GRC/MBIC_50_	MIC (mg/L)
*E. coli*	ATCC 25922	1000	50	100	200	20	> 1200
	K-12	800	50–100	100	100	8–16	> 1200
	O157:H7	1000	25–50	50	100	20–40	> 1200
	DH5α	1200	50	100	100	24	> 1200
PAO1		800	600–800	800	800	1.0–1.3	> 1200
MRSA ATCC 43300		500	100–200	200	200	2.5–5	> 1200

Results are representative of at least three independent experiments performed in triplicates

^a^The GRC (growth-retarding concentration) is the concentration needed to decrease the OD_595_ by more than 30% compared to that of a negative control after 16 h of incubation during MIC assay except for PAO1 whose GRC was determined after 12 h. There is no endpoint of MIC in this study

^b^The MBIC_50_ is the concentration resulting in a 50% OD_550_ reduction in metabolic activity of viable cells in remained biofilm compared to that of an untreated control in MBIC assay

### BU Effectively Inhibits Biofilm Formation and Eradicates Pre-formed Biofilm

BU inhibited biofilm formation and eradicated mature biofilms of all the six tested strains (Fig. 1, Fig. S3, and Table 1). MBIC values of BU were 50 mg/L for *E. coli* O157:H7; 100 mg/L for *E. coli* ATCC 25922, *E. coli* K-12, and *E. coli* DH5α; 200 mg/L for MRSA; and 800 mg/L for PAO1, respectively. MBEC values were 100 mg/L for *E. coli* K-12, *E. coli* O157:H7, and *E. coli* DH5α; 200 mg/L for *E. coli* ATCC 25922 and MRSA; and 800 mg/L for PAO1, respectively. For *E. coli* strains and MRSA, all MBIC and MBEC values (50–200 mg/L) were lower than the MIC values (> 1200 mg/L) and the GRC values (500–1200 mg/L), suggesting bacterial growth or survival might only be mildly affected under the effective antibiofilm concentrations of BU (Table 1). Furthermore, when treated with BU at concentrations exceeding 400 mg/L, biofilms of PAO1 presented a surficial tears phenomenon (Fig. S4), which might make bacterial cells more readily removed by blood flow if there is any. These results indicated that BU was effective in inhibiting biofilm formation and eradicating pre-formed biofilms.

### BU Decreases Both Biofilm Coverage and Thickness, and Destroys Biofilm Matrix

**Fig. 2:**
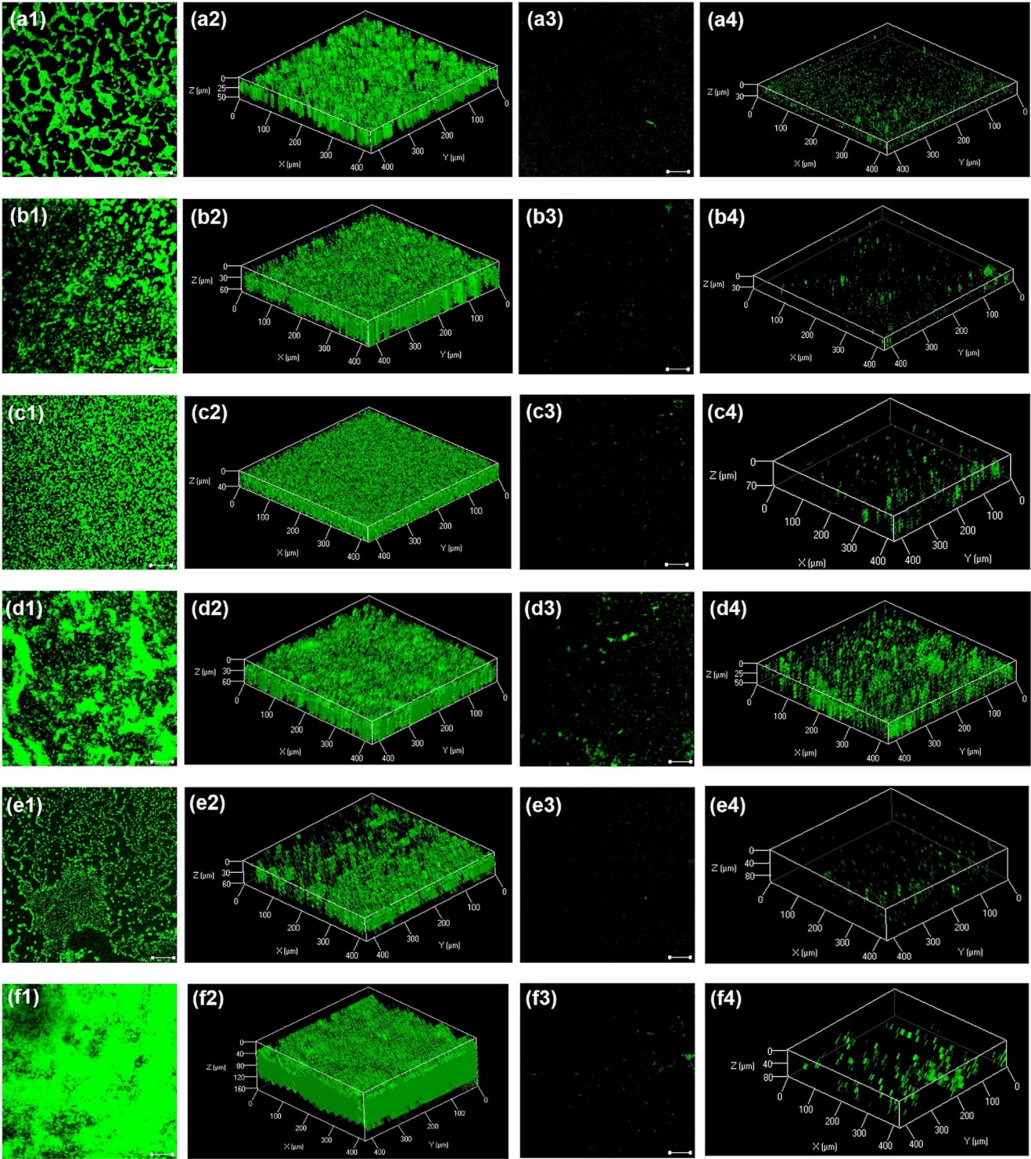
CLSM analyses of biofilm formation inhibitory effects of butenolide (BU). (a) *E. coli* ATCC 25922. (b) *E. coli* K-12. (c) *E. coli* O157:H7. (d) *E. coli* DH5α. (e) strain PAO1. (f) MRSA strain ATCC 43300. Series 1 and 2 were biofilms after 24 h incubation without BU treatment. Series 3 and 4 were biofilms after 24 h incubation treated with BU under MBICs (100 mg/L for *E. coli* K-12, ATCC 25922, and DH5α; 50 mg/L for *E. coli* O157:H7; 800 mg/L for PAO1; 200 mg/L for MRSA). Dyes of SYTO-9 and propidium iodide, wavelength of 488 and 561 nm, and ×20 magnification were used to observe. Scale bar is 50 μm

**Fig. 3:**
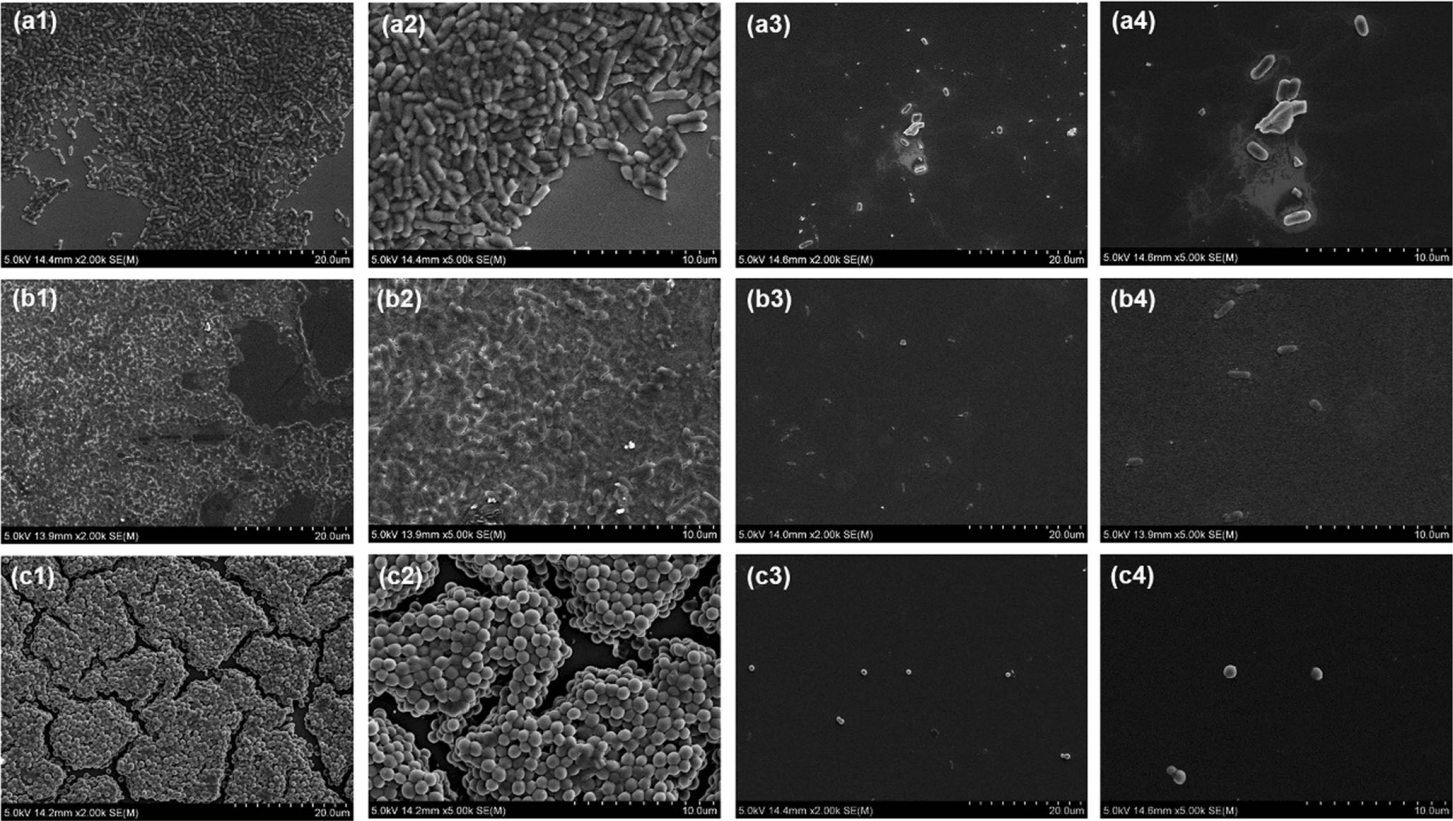
SEM analyses of the effects of butenolide (BU) on biofilm structure. (a) *E. coli* K-12. (b) strain PAO1. (c) MRSA strain ATCC 43300. Series 1 and 2 were biofilms after 24 h incubation without BU treatment, with ×2000 and ×5000 magnification, respectively. Series 3 and 4 were biofilms after 24 h incubation treated with BU under MBICs (100 mg/L for *E. coli* K-12; 800 mg/L for PAO1; 200 mg/L for MRSA), with ×2000 and ×5000 magnification, respectively. Complex and intact biofilm matrix were visible without BU treatment

To investigate the effects of BU on biofilm structures, CLSM and SEM were employed to assess structural features of biofilms treated with MBICs of BU. All untreated strains formed biofilms with unambiguous EPSs and intact structures (Figs. 2 and 3). In contrast, biofilms treated with BU had different extents of biofilm removal. The quantitative results from CLSM showed that biofilm average coverage, thickness, and biovolume of the six strains were decreased by over 90% compared to the untreated counterparts (Table 2). The effects of BU on biofilm structures of *E. coli* K-12, PAO1, and MRSA were visualized by SEM. No intact biofilms or embedded bacterial cells were visible under MBICs of BU (Fig. 3). Since MBIC of BU for PAO1 was the same to its GRC (800 mg/L), antibiofilm effect of BU against biofilm of PAO1 was also tested at 200 mg/L. Figure S5 showed that 200 mg/L of BU reduced by 68% of biofilm average coverage and 80% of biofilm thickness compared to negative control. These results indicated that BU could greatly reduce *P. aeruginosa* biofilm formation.

**Table 2:** COMSTAT quantitative analyses of biofilms exposure to butenolide (BU) under MBICs

Strain		Average coverage (%)		Average Thickness (μm)		Biovolume (μm^3^/μm^2^)	
		Control	MBIC	Control	MBIC	Control	MBIC
*E. coli*	ATCC 25922	19.12 ± 1.68	2.62 ± 1.41	29.16 ± 3.13	0.65 ± 0.34	12.84 ± 1.33	0.07 ± 0.01
	K-12	18.81 ± 4.36	1.38 ± 0.55	27.94 ± 6.80	0.16 ± 0.07	18.00 ± 2.51	0.01 ± 0.00
	O157:H7	17.27 ± 3.68	1.56 ± 0.69	38.42 ± 6.84	0.23 ± 0.10	19.90 ± 2.68	0.40 ± 0.08
	DH5α	19.88 ± 4.41	1.74 ± 0.86	45.22 ± 7.37	3.98 ± 1.19	30.09 ± 4.87	0.41 ± 0.12
PAO1		20.82 ± 1.81	2.52 ± 0.59	6.52 ± 1.69	1.18 ± 0.93	8.94 ± 1.65	0.09 ± 0.01
MRSA ATCC 43300		52.11 ± 8.45	0.23 ± 0.17	71.56 ± 11.58	0.66 ± 0.53	117.27 ± 22.38	0.01 ± 0.00

Data obtained from COMSTAT analyses of Z-stack images of biofilms acquired through CLSM. Values expressed as mean ± standard deviation

### BU May Be a Nonspecific QS Inhibitor

At concentrations of 5, 12.5, and 25 mg/L, BU could reduce luminescence of *V. harveyi* BB170 by ~ 25, ~ 50, and over 70%, respectively (Fig. S6a). However, BU at concentrations above 12.5 mg/L caused growth inhibition to the bacterial cells (Fig. S6b). Same as the effect of BU on AI-2 mediated QS system, BU inhibited short-chain AHLs at a concentration of 100 mg/L and long-chain AHLs at 50 mg/L (Fig. S7a) while a growth inhibition of ~ 20% was observed in both reporter strains at concentrations of 25–50 mg/L (Fig. S7b and Fig. S7c). These results indicated that BU could be a nonspecific QS inhibitor which influences both AI-2 and AHLs mediated QS in reporter strains through interfering with bacterial growth.

### BU Could Be a Tetracycline Enhancer

Initially, the pairs of antibiotics were screened for their drug interaction, defined as FICI, where FICI ≤ 0.5 indicates synergy, 0.5 < FICI ≤ 1.0 indicates additive, 1.0 < FICI ≤ 4.0 indicates no interaction, and FICI > 4.0 indicates antagonism (Odds 2003). The FICIs between BU and tetracycline (Table 3) against biofilms of *E. coli* O157:H7, PAO1, and MRSA were 0.5, 0.25, and 0.5, respectively, indicating synergistic effect between two compounds against these bacterial biofilms. Compared to tetracycline alone, the MBECs of tetracycline in combination with BU decreased by 75% for *E. coli* O157:H7 and MRSA, as well as 87.5% for PAO1. For the other three strains, *E. coli* ATCC 25922, K-12, and DH5α, the FICIs ranged from 0.5 to 1.0, suggesting additive effects of BU with tetracycline. These results indicated that BU had the potential to act alone, or to work with commonly used antibiotics in a synergistic/enhancing manner against biofilm infections.

**Table 3:** Drug interactions of butenolide (BU) and tetracycline against 24 h pre-formed biofilms in a modified chequerboard assay

Strain		MBEC (mg/L)			FICI^a^
		BU	Tetracycline	Combination	
*E. coli*	ATCC 25922	200	20	100 + 1.25	0.563
	K-12	100	4	50 + 1	0.75
	O157:H7	100	4	25 + 1	0.5
	DH5α	100	2	25 + 1.25	0.875
PAO1		800	80	100 + 10	0.25
MRSA ATCC 43300		200	200	50 + 50	0.5

^a^FICI: the fractional inhibitory concentration index, drugs interaction was synergistic when FICI ≤ 0.5, additive when 0.5 < FICI ≤ 1.0, indifferent when 1.0 < FICI ≤ 4.0, and antagonistic when FICI > 4

### The Lipophilicity Plays an Essential Role in Antibiofilm Activity of BU

**Fig. 4:**
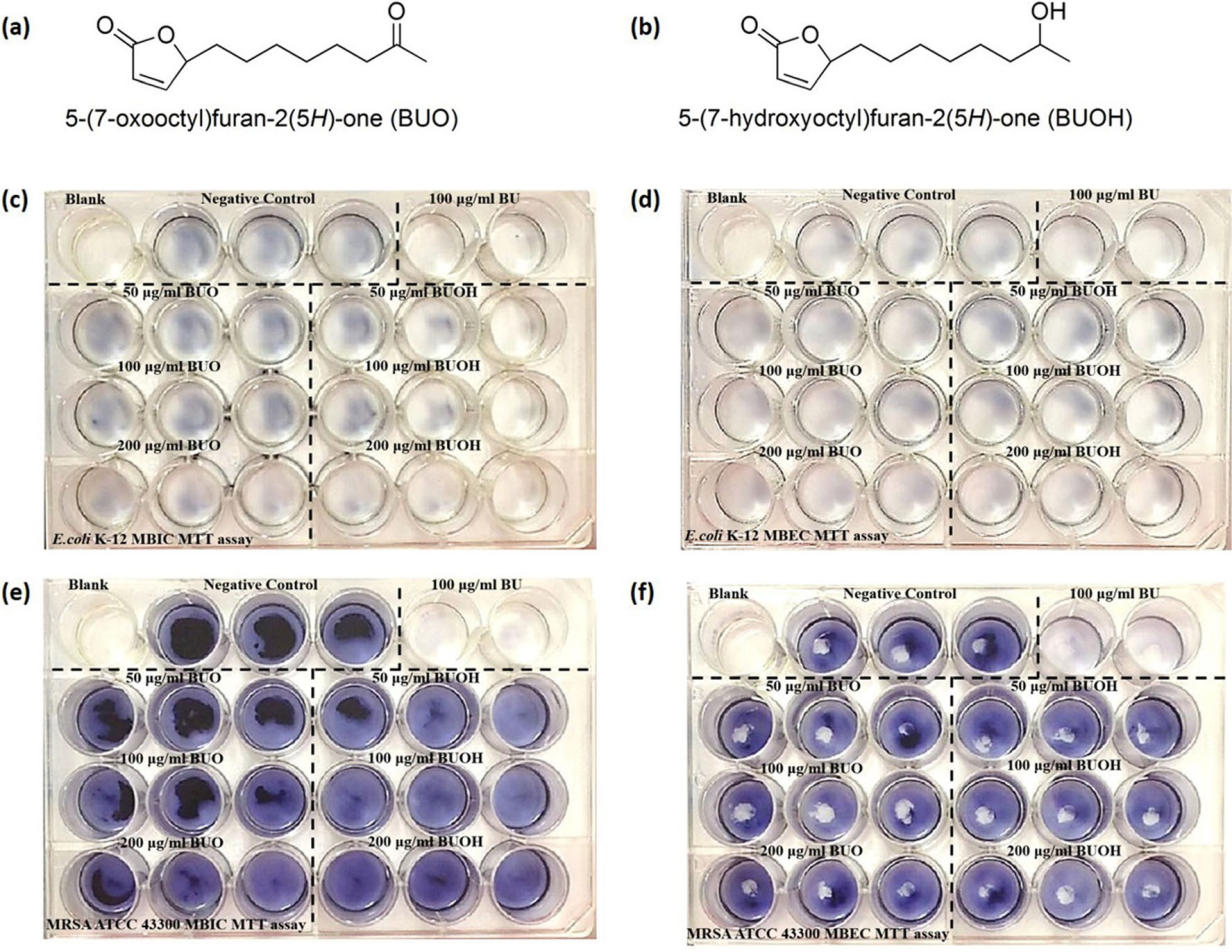
Two high hydrophilic analogs of butenolide (BU), BUO and BUOH, and their antibiofilm activity against *E. coli* K-12 and MRSA ATCC 43300 biofilms. Antibiofilm abilities of MBIC and MBEC (24 h pre-formed biofilm) were investigated. DMSO is referred as negative control, and BU of MBIC or MBEC (100 mg/L for *E. coli* K-12 and 200 mg/L for MRSA ATCC 43300) is referred as positive control. **a** Chemical structure of BUO with a carbonyl on the seven carbon in alkyl side-chain of BU. **b** Chemical structure of BUOH with a hydroxyl on the seven carbon in alkyl side-chain of BU. **c**, **d** MBIC and MBEC assay of both BUO and BUOH on *E. coli* K-12 biofilm inhibition and eradication. **e**, **f** MBIC and MBEC assay of both BUO and BUOH on MRSA ATCC 43300 biofilm inhibition and eradication. No antibiofilm activities of BUO and BUOH were observed

As shown in Fig. 4, both BOU and BOUH (structures shown in Fig. 4a, b) are hydrophilic structural analogs of BU. These two analogs were only low toxic to tested strains (Fig. S8); however, neither of them exhibited any activity to inhibit or to eradicate biofilms. These results indicated that strong hydrophilicity might hinder the penetration of compounds into the EPSs matrix of biofilms. In contrast, strong lipophilicity ensures effective diffusion of BU into cells to reach target molecules. As such, lipophilicity plays an important role in antibiofilm activity of BU.

## Discussion

Butenolide (abbreviated as BU) and its analogs have previously been reported as a promising and broad-range antifouling agent (Li et al. 2012; Xu et al. 2010; Zhang et al. 2012). More recent studies showed that BU also has effective antibiofilm activities against multi-species biofilm formation in natural marine environment (Ding et al. 2018). The structural similarity between BU and furanones suggested that BU might have the bioactivity against pathogenic biofilms. Different laboratories focused on the effects of different derivatives of brominated furanones against different biofilms, making it difficult to compare their antibiofilm activities for further use (He et al. 2012; Hentzer et al. 2002; Lönn-Stensrud et al. 2008; Lönn-Stensrud et al. 2007; Ren et al. 2002; Ren et al. 2001; Wu et al. 2015b). So far, a single furanone targeting a broad spectrum of bacteria has not been discovered. In the present study, a more comprehensive antibiofilm comparison between BU and previously reported brominated furanones (from 2000 to 2016) was made, for better understanding about antibiofilm activities of different chemicals.

Based on the comparisons of chemical structures, antibiofilm activities, and targeted biofilm strains between BU from our work and brominated furanones from the literature (summarized in Table S1), the following points can be made to illustrate the antibiofilm potential of BU in clinical application. First, the MBIC values and inhibitory rates of all brominated furanones have not been specified and MBEC values were seldomly determined. Zhao et al. even reported that furanone C-30 had no eradication activity against pre-formed biofilm of *Acidithiobacillus ferrooxidans* (Zhao et al. 2015). In fact, antibiofilm activity should include two aspects: the ability to inhibit initial biofilm formation and to eradicate existing mature biofilm. Inhibition of initial biofilm formation is definitely effective in treating microbial infections, but eradication of the existing biofilm, which causes serious chronic and recurrent infections (Høiby 2011; Høiby et al. 2010; Wu et al. 2015a), is equally important. However, the index of eradicating existing biofilm was generally ignored in previous brominated furanones-related studies. In the present study, BU can both inhibit biofilm formation and eradicate existing biofilms, making it a promising candidate to treat biofilm infections.

Secondly, a conjugated exocyclic vinyl bromide on the furanone ring was critical to antibiofilm activity of brominated furanones (Han et al. 2008; Janssens et al. 2008; Lönn-Stensrud et al. 2008), but halogen elements made brominated furanones toxic to mammalian cells (Rabin et al. 2015). Our results confirmed that 2-furanone ring was the core structure to these analogs consistently, but halogen elements were not indispensable for antibiofilm activity of furanones. In addition, we found the lipophilicity of the compound also greatly affects its activity. BU is highly lipophilic due to the 8-carbon alkyl side-chain. On the contrary, both hydrophilic analogs of BU (e.g., BUO and BUOH, Fig. 4) lost the ability against bacterial biofilm. These results demonstrated that 2-furanone ring and strong lipophilicity played important roles in the efficient antibiofilm activity of BU.

Thirdly, as compared to brominated furanones, BU is a broad-spectrum antibiofilm agent that effectively inhibited both Gram-negative and Gram-positive bacterial biofilms. Additionally, in the present study, GRC/MBIC_50_ was calculated (Tables 1 and S1) as a comprehensive and standard way for cross comparison of similar chemicals’ antibiofilm activities. The GRC/MBIC_50_ values of BU against *E. coli* ATCC 25922, K-12, O157:H7, and DH5α were 20, 8–16, 20–40, and 24, respectively, which were much higher (40 to 200 times) than those of natural furanones against *E. coli* biofilms. In regard of rapid development of drug resistance, BU should be safer than brominated furanones in long-term treatments as a smaller selective pressure is posed to the bacteria.

The brominated furanones are potent antibiofilm agents whose mechanism of action has been largely attributed to their capability to inhibit QS processes in bacteria (Brackman and Coenye 2015; Rice et al. 2005). The similar chemical structure between brominated furanones and BU also promoted us to conduct some preliminary tests to investigate the effects of BU on QS systems. As shown in Fig. S6 and Fig. S7, BU could inhibit AI-2 and four types of AHLs-mediated QS system to a great extent; however, it also exhibited ~ 20% growth inhibition to QS reporter strains under concentration of 50 mg/L. Regarding this, BU could influence the QS system but QS might not be its primary target. More molecular experiments need to be carried out to elucidate the antibiofilm mechanism of BU in the future.

After nearly two decades of investigations, antibiofilm substances can be divided into two general groups, namely antibacterial and antibiofilm substances. The antibacterial group includes antibiotics such as antibiotic analogs, antibacterial peptides, and antibacterial glycolipids (Dusane et al. 2011; Luca et al. 2013; Luo et al. 2017; Nair et al. 2016; Ooi et al. 2016). For examples, a glycolipid derived from *Serratia marcescens* was active against *Candida albica* and *P. aeruginosa* biofilms, with 100 mg/L of MBECs but only 25 mg/L of MICs (Dusane et al. 2011). A bacterial protein P128 was effective against biofilms of several *S. aureus* strains, with 200–1000 mg/L of MBECs but only 4–8 mg/L of MICs (Nair et al. 2016). A amphibian peptide Esc(1-21) was effective against biofilms of *P. aeruginosa* strains, with 12–48 mg/L of MBECs but only 4–8 mg/L of MICs (Luca et al. 2013). As suggested by their MIC/MBEC values, these compounds actually inhibit or eradicate biofilms simply due to their bactericidal effects, so that rapid development of drug resistance would be a common response from bacteria. In contrast, the antibiofilm group has no or low antibacterial activity, thus has the potential to be applied in long-term clinical treatments. Recently, Devi et al. reported a 70% of *Aeromonas hydrophila* biofilm reduction when treated with 1000 mg/L of rosmarinic acid (Rama Devi et al. 2016). In another study, promethazine is active against biofilms of *Burkholderia pseudomallei* (MBECs = 780–3120 mg/L) (Sidrim et al. 2017). However, efficient antibiofilm compounds without bactericidal activities are rare (Rabin et al. 2015). Considering biofilms promote rapid development of antibiotic resistance, there is an urgent need for exploring efficient antibiofilm agents for combinational drug therapy.

The FICI is one of most frequently used measurements of synergistic effects between different drugs. The FICIs of BU with tetracycline against biofilms of *E. coli* O157:H7, PAO1, and MRSA ranged from 0.25 to 0.5, indicating BU’s potential to act as an enhancer to promote efficacy of antibiotics and to reduce the emergence of drug resistance. Therefore, BU may be able to act as a pioneer molecule to penetrate and destroy biofilm matrix followed by antibiotic treatments against serious biofilm related infections.

To summarize, we have demonstrated for the first time that BU had promising antibiofilm activity and low antibacterial activity against different types of pathogens (both Gram-positive and Gram-negative bacteria). More importantly, BU effectively inhibited biofilm formation and eradicated pre-formed biofilm. In addition, the lipophilicity of BU was critical to its antibiofilm activity. Our work illustrates that BU is a promising antibiofilm agent and antibiotics enhancer, which may hold a great potential in future applications.

## Electronic Supplementary Material

ESM 1 (DOCX 5876 kb)

## References

[CR1] Brackman G, Coenye T (2015). Quorum sensing inhibitors as anti-biofilm agents. Curr Pharm Des.

[CR2] Bren A, Park JO, Towbin BD, Dekel E, Rabinowitz JD, Alon U (2016). Glucose becomes one of the worst carbon sources for *E. coli* on poor nitrogen sources due to suboptimal levels of cAMP. Sci Rep.

[CR3] Bryers JD (2008). Medical biofilms. Biotechnol Bioeng.

[CR4] Castelo-Branco D, Riello GB, Vasconcelos DC, Guedes GMM, Serpa R, Bandeira T, Monteiro AJ, Cordeiro RA, Rocha MFG, Sidrim JJC, Brilhante RSN (2016). Farnesol increases the susceptibility of *Burkholderia pseudomallei* biofilm to antimicrobials used to treat melioidosis. J Appl Microbiol.

[CR5] Clinical and Laboratory Standards Institute (2015). Performance standards for antimicrobial susceptibility testing: twenty-fifth informational supplement.

[CR6] Costerton JW, Stewart PS, Greenberg EP (1999). Bacterial biofilms: a common cause of persistent infections. Science.

[CR7] de la Fuente-Nunez C, Reffuveille F, Fernandez L, Hancock REW (2013). Bacterial biofilm development as a multicellular adaptation: antibiotic resistance and new therapeutic strategies. Curr Opin Microbiol.

[CR8] de Nys R, Wright AD, König GM, Sticher O (1993). New halogenated furanones from the marine alga *Delisea pulchra* (cf. *fimbriata*). Tetrahedron.

[CR9] Ding W, Ma C, Zhang W, Chiang H, Tam C, Xu Y, Zhang G, Qian P-Y (2018). Anti-biofilm effect of a butenolide/polymer coating and metatranscriptomic analyses. Biofouling.

[CR10] Dusane DH, Pawar VS, Nancharaiah YV, Venugopalan VP, Kumar AR, Zinjarde SS (2011). Anti-biofilm potential of a glycolipid surfactant produced by a tropical marine strain of *Serratia marcescens*. Biofouling.

[CR11] Francolini I, Donelli G (2010). Prevention and control of biofilm-based medical-device-related infections. FEMS Immunol Med Microbiol.

[CR12] Fux C, Costerton JW, Stewart PS, Stoodley P (2005). Survival strategies of infectious biofilms. Trends Microbiol.

[CR13] Gilbert P, Allison D, McBain A (2002). Biofilms in vitro and in vivo: do singular mechanisms imply cross-resistance?. J Appl Microbiol.

[CR14] Hall-Stoodley L, Costerton JW, Stoodley P (2004). Bacterial biofilms: from the natural environment to infectious diseases. Nat Rev Microbiol.

[CR15] Han Y, Hou S, Simon KA, Ren DC, Luk Y-Y (2008). Identifying the important structural elements of brominated furanones for inhibiting biofilm formation by *Escherichia coli*. Bioorg Med Chem Lett.

[CR16] He Z, Wang Q, Hu Y, Liang J, Jiang Y, Ma R, Tang Z, Huang Z (2012). Use of the quorum sensing inhibitor furanone C-30 to interfere with biofilm formation by *Streptococcus mutans* and its *luxS* mutant strain. Int J Antimicrob Agents.

[CR17] Hentzer M, Riedel K, Rasmussen TB, Heydorn A, Andersen JB, Parsek MR, Rice SA, Eberl L, Molin S, Høiby N, Kjelleberg S, Givskov M (2002). Inhibition of quorum sensing in *Pseudomonas aeruginosa* biofilm bacteria by a halogenated furanone compound. Microbiology.

[CR18] Hentzer M, Wu H, Andersen JB, Riedel K, Rasmussen TB, Bagge N, Kumar N, Schembri MA, Song Z, Kristoffersen P (2003). Attenuation of *Pseudomonas aeruginosa* virulence by quorum sensing inhibitors. EMBO J.

[CR19] Heydorn A, Nielsen AT, Hentzer M, Sternberg C, Givskov M, Ersbøll BK, Molin S (2000). Quantification of biofilm structures by the novel computer program COMSTAT. Microbiology.

[CR20] Høiby N (2011). Recent advances in the treatment of *Pseudomonas aeruginosa* infections in cystic fibrosis. BMC Med.

[CR21] Høiby N, Bjarnsholt T, Givskov M, Molin S, Ciofu O (2010). Antibiotic resistance of bacterial biofilms. Int J Antimicrob Agents.

[CR22] Høiby N, Ciofu O, Johansen HK, Song Z-J, Moser C, Jensen PØ, Molin S, Givskov M, Tolker-Nielsen T, Bjarnsholt T (2011). The clinical impact of bacterial biofilms. Int J Oral Sci.

[CR23] Hume E, Baveja J, Muir B, Schubert T, Kumar N, Kjelleberg S, Griesser HJ, Thissen H, Read R, Poole-Warren L (2004). The control of *Staphylococcus epidermidis* biofilm formation and in vivo infection rates by covalently bound furanones. Biomaterials.

[CR24] Janssens JCA, Steenackers H, Robijns S, Gellens E, Levin J, Zhao H, Hermans K, Coster DD, Verhoeven TL, Marchal K (2008). Brominated furanones inhibit biofilm formation by *Salmonella enterica* serovar *Typhimurium*. Appl Environ Microbiol.

[CR25] Kolter R (2010). Biofilms in lab and nature: a molecular geneticist’s voyage to microbial ecology. Int Microbiol.

[CR26] Kuehl R, Albataineh S, Gordon O, Luginbuehl R, Otto M, Textor M, Landmann R (2009). Furanone at subinhibitory concentrations enhances staphylococcal biofilm formation by *luxS* repression. Antimicrob Agents Chemother.

[CR27] Lasarre B, Federle MJ (2013). Exploiting quorum sensing to confuse bacterial pathogens. Microbiol Mol Biol Rev.

[CR28] Lewis K (2006). Persister cells, dormancy and infectious disease. Nat Rev Microbiol.

[CR29] Lewis K (2010). Persister Cells. Annu Rev Microbiol.

[CR30] Li Y, Zhang F, Xu Y, Matsumura K, Han Z, Liu L, Lin W, Jia Y, Qian P-Y (2012). Structural optimization and evaluation of butenolides as potent antifouling agents: modification of the side chain affects the biological activities of compounds. Biofouling.

[CR31] Lönn-Stensrud J, Petersen F, Benneche T, Scheie AA (2007). Synthetic bromated furanone inhibits autoinducer-2-mediated communication and biofilm formation in oral streptococci. Mol Oral Microbiol.

[CR32] Lönn-Stensrud J, Landin MA, Benneche T, Petersen FC, Scheie AA (2008). Furanones, potential agents for preventing *Staphylococcus epidermidis* biofilm infections?. J Antimicrob Chemother.

[CR33] Luca V, Stringaro A, Colone M, Pini A, Mangoni ML (2013). Esculentin(1-21), an amphibian skin membrane-active peptide with potent activity on both planktonic and biofilm cells of the bacterial pathogen *Pseudomonas aeruginosa*. Cell Mol Life Sci.

[CR34] Luo Y, McLean DTF, Linden GJ, McAuley DF, McMullan R, Lundy FT (2017). The naturally occurring host defense peptide, LL-37, and its truncated mimetics KE-18 and KR-12 have selected biocidal and antibiofilm activities against *Candida albicans*, *Staphylococcus aureus*, and *Escherichia coli* in vitro. Front Microbiol.

[CR35] Manefield M, Rasmussen TB, Henzter M, Andersen JB, Steinberg P, Kjelleberg S, Givskov M (2002). Halogenated furanones inhibit quorum sensing through accelerated LuxR turnover. Microbiology.

[CR36] Martinelli D, Grossmann G, Séquin U, Brandl H, Bachofen R (2004). Effects of natural and chemically synthesized furanones on quorum sensing in *Chromobacterium violaceum*. BMC Microbiol.

[CR37] McClean KH, Winson MK, Fish L, Taylor A, Chhabra SR, Camara M, Daykin M, Lamb JH, Swift S, Bycroft BW (1997). Quorum sensing and *Chromobacterium violaceum*: exploitation of violacein production and inhibition for the detection of *N*-acylhomoserine lactones. Microbiology.

[CR38] Nair S, Desai S, Poonacha N, Vipra A, Sharma U (2016). Antibiofilm activity and synergistic inhibition of *Staphylococcus aureus* biofilms by bactericidal protein P128 in combination with antibiotics. Antimicrob Agents Chemother.

[CR39] National Institutes of Health (2002) Data from “Research on microbial biofilms (PA-03-047)”. National Heart, Lung, and Blood Institute https://grants.nih.gov/grants/guide/pa-files/PA-03-047.html. Accessed 1 March 2017

[CR40] O’Toole G, Kaplan HB, Kolter R (2000). Biofilm formation as microbial development. Annu Rev Microbiol.

[CR41] Odds FC (2003). Synergy, antagonism, and what the chequerboard puts between them. J Antimicrob Chemother.

[CR42] Ooi N, Eady EA, Cove JH, O'Neill AJ (2016). Tert-butyl benzoquinone: mechanism of biofilm eradication and potential for use as a topical antibiofilm agent. J Antimicrob Chemother.

[CR43] Rabin N, Zheng Y, Opoku-Temeng C, Du Y, Bonsu E, Sintim HO (2015). Agents that inhibit bacterial biofilm formation. Future Med Chem.

[CR44] Rama Devi K, Srinivasan R, Kannappan A, Santhakumari S, Bhuvaneswari M, Rajasekar P, Prabhu NM, Veera Ravi A (2016). In vitro and in vivo efficacy of rosmarinic acid on quorum sensing mediated biofilm formation and virulence factor production in *Aeromonas hydrophila*. Biofouling.

[CR45] Ramritu P, Halton K, Collignon P, Cook D, Fraenkel D, Battistutta D, Whitby M, Graves N (2008). A systematic review comparing the relative effectiveness of antimicrobial-coated catheters in intensive care units. Am J Infect Control.

[CR46] Ren DC, Sims JJ, Wood TK (2001). Inhibition of biofilm formation and swarming of *Escherichia coli* by (*5Z*)-4-bromo-5-(bromomethylene)-3-butyl-2(*5H*)-furanone. Environ Microbiol.

[CR47] Ren DC, Sims JJ, Wood TK (2002). Inhibition of biofilm formation and swarming of *Bacillus subtilis* by (*5Z*)-4-bromo-5-(bromomethylene)-3-butyl-2(*5H*)-furanone. Lett Appl Microbiol.

[CR48] Ren DC, Bedzyk LA, Thomas SM, Ye RW, Wood TK (2004). Gene expression in *Escherichia coli* biofilms. Appl Microbiol Biotechnol.

[CR49] Ren DC, Bedzyk LA, Ye RW, Thomas SM, Wood TK (2004). Differential gene expression shows natural brominated furanones interfere with the autoinducer-2 bacterial signaling system of *Escherichia coli*. Biotechnol Bioeng.

[CR50] Rice SA, McDougald D, Kumar N, Kjelleberg S (2005). The use of quorum-sensing blockers as therapeutic agents for the control of biofilm-associated infections. Curr Opin Investig Drugs.

[CR51] Sidrim JJC, Vasconcelos DC, Riello GB, Guedes GMDM, Serpa R, Bandeira TDJPG, Monteiro AJ, Cordeiro RDA, Castelo-Branco DDSCM, Rocha MFG, Brilhante RSN (2017). Promethazine improves antibiotic efficacy and disrupts biofilms of *Burkholderia pseudomallei*. Biofouling.

[CR52] Someya N, Morohoshi T, Okano N, Otsu E, Usuki K, Sayama M, Sekiguchi H, Ikeda T, Ishida S (2009). Distribution of *N*-acylhomoserine lactone-producing fluorescent pseudomonads in the phyllosphere and rhizosphere of potato (*Solanum tuberosum* L.). Microbes Environ.

[CR53] Taga ME, Xavier KB (2005) Methods for analysis of bacterial autoinducer-2 production. Curr Protoc Microbiol 23(1). Chapter 1: Unit1C.1. 10.1002/9780471729259.mc01c01s23 10.1002/9780471729259.mc01c01s23 22045583

[CR54] Waters CM, Bassler BL (2005). Quorum sensing: cell-to-cell communication in bacteria. Annu Rev Cell Dev Biol.

[CR55] Wu H, Moser C, Wang H-Z, Høiby N, Song Z-J (2015). Strategies for combating bacterial biofilm infections. Int J Oral Sci.

[CR56] Wu Y, Quan X, Si X (2015). Incorporation of brominated furanone into Nafion polymer enhanced anti-biofilm efficacy. Int Biodeter Biodegr.

[CR57] Xu Y, He H, Schulz S, Liu X, Fusetani N, Xiong H, Xiao X, Qian P-Y (2010). Potent antifouling compounds produced by marine *Streptomyces*. Bioresour Technol.

[CR58] Zhang Y-F, Zhang H, He L, Liu C, Xu Y, Qian P-Y (2012). Butenolide inhibits marine fouling by altering the primary metabolism of three target organisms. ACS Chem Biol.

[CR59] Zhao Y, Chen P, Nan W, Zhi D, Liu R, Li H (2015). The use of (5Z)-4-bromo-5-(bromomethylene)-2(5H)-furanone for controlling acid mine drainage through the inhibition of *Acidithiobacillus ferrooxidans* biofilm formation. Bioresour Technol.

