# Supplementary material for: Butenolide, a Marine-Derived Broad-Spectrum Antibiofilm Agent Against Both Gram-Positive and Gram-Negative Pathogenic Bacteria
Source: Mar Biotechnol (NY). 2019 Jan 5;21(1):88–98. doi: 10.1007/s10126-018-9861-1 (PMC6394721; doi:10.1007/s10126-018-9861-1)
Supplement: Supplementary file 1 — (DOCX 5876 kb) [file 10126_2018_9861_MOESM1_ESM.docx]

**Supplementary data**

**Butenolide, a marine derived broad-spectrum antibiofilm agent against both Gram-positive and Gram-negative pathogenic bacteria**

Qi Yin^1,2^, Jinyou Liang^1^, Weipeng Zhang^3^, Lv Zhang^1^, Zhang-Li Hu^1^, Yu Zhang^1^*, Ying Xu^1^*

*Marine Biotechnology*

^a^Shenzhen Key Laboratory of Marine Bioresource and Eco-environmental Science, Shenzhen Engineering Laboratory for Marine Algal Biotechnology, College of Life Sciences and Oceanography, Shenzhen University, Shenzhen 518060, P.R. China

^b^State Key Laboratory of Biotherapy / Collaborative Innovation Center for Biotherapy, West China Hospital, West China Medical School, Sichuan University; No. 17, Section 3, South Renmin Road, Chengdu, Sichuan, 610041, P.R. China

^c^Division of Life Science, Hong Kong University of Science and Technology, Hong Kong SAR, P.R. China

***Address correspondence to**:

College of Life Sciences and Oceanography, Shenzhen University, 3688 Nanhai Avenue, Nanshan Section, Shenzhen 518060, PR China

Tel.: +86 755 26958849; fax: +86 755 26958849.

Email address: [boxuying@szu.edu.cn](mailto:boxuying@szu.edu.cn); [biozy@szu.edu.cn](mailto:biozy@szu.edu.cn)


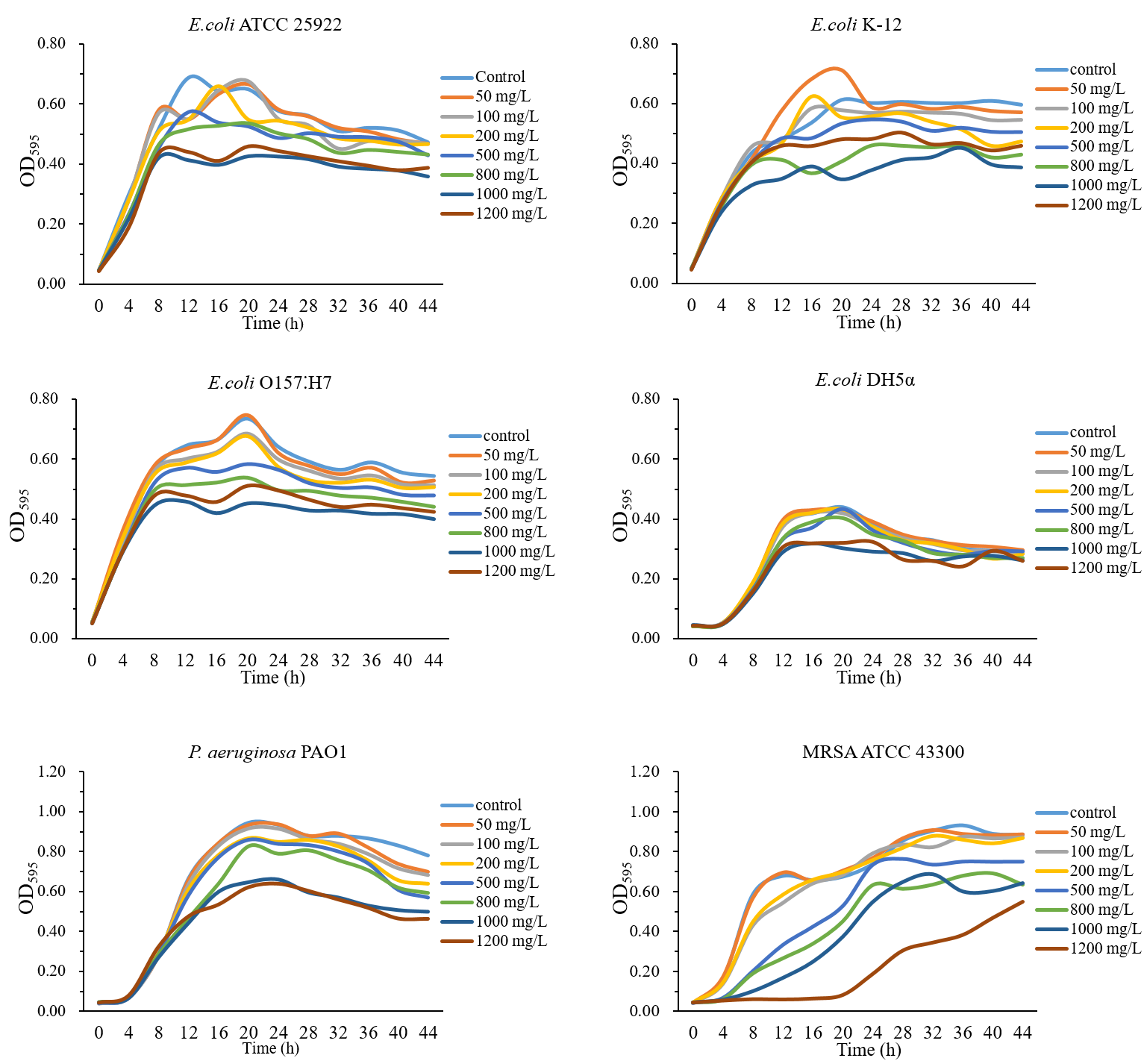


**Fig. S1** Growth curves of six strains treated with various concentrations (50 mg/L, 100 mg/L, 200 mg/L, 500 mg/L, 800 mg/L, 1000 mg/L, or 1200 mg/L) of butenolide. The experiments were performed in triplicates and repeated three times.


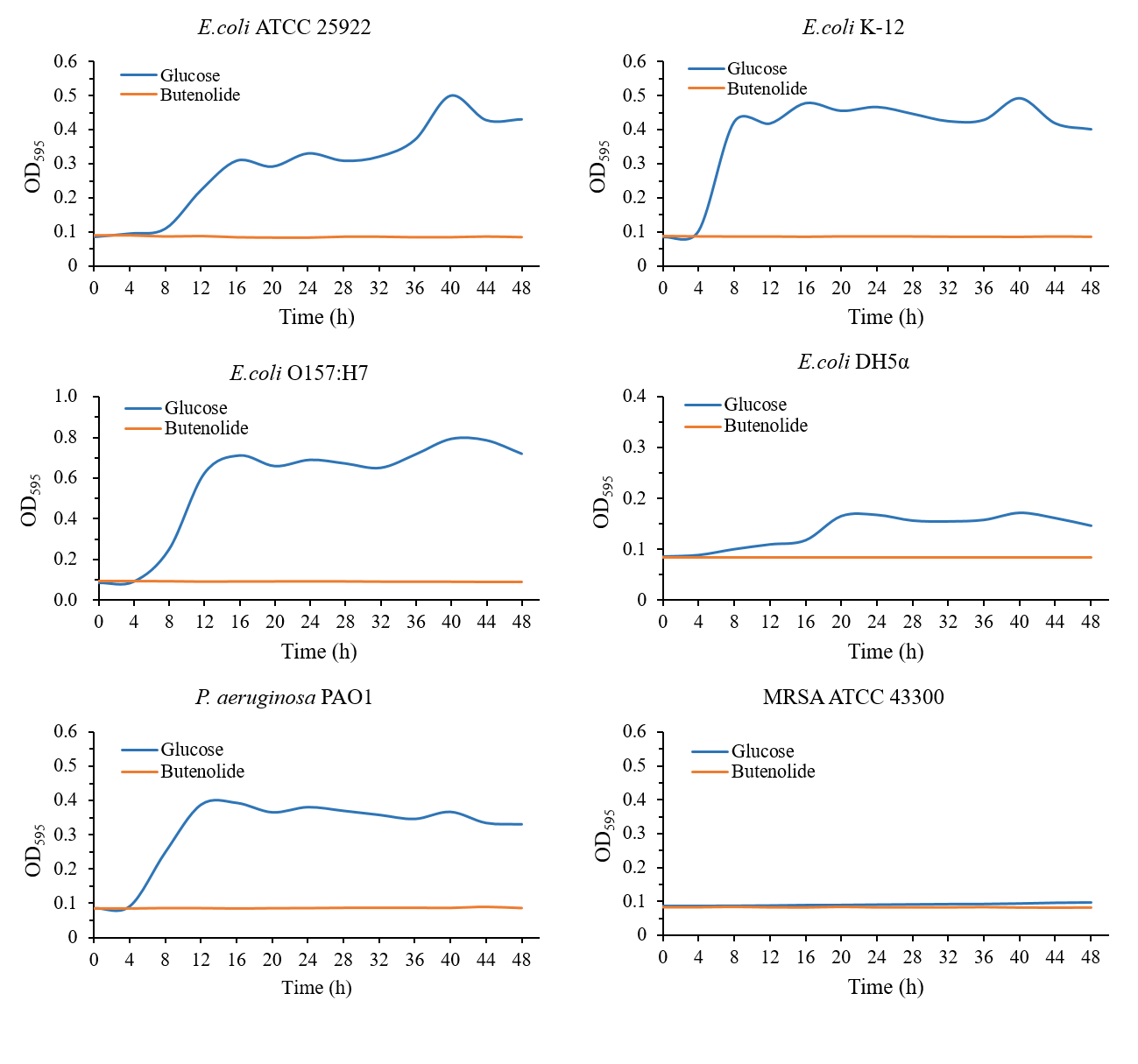


**Fig. S2** Growth cruves of six strain using equimolar carbon (66 mM) glucose and butenolide (BU) as sole carbon sourse. The experiments were performed in triplicates and repeated three times. All six tested strains could ont use BU as sole carbon source.


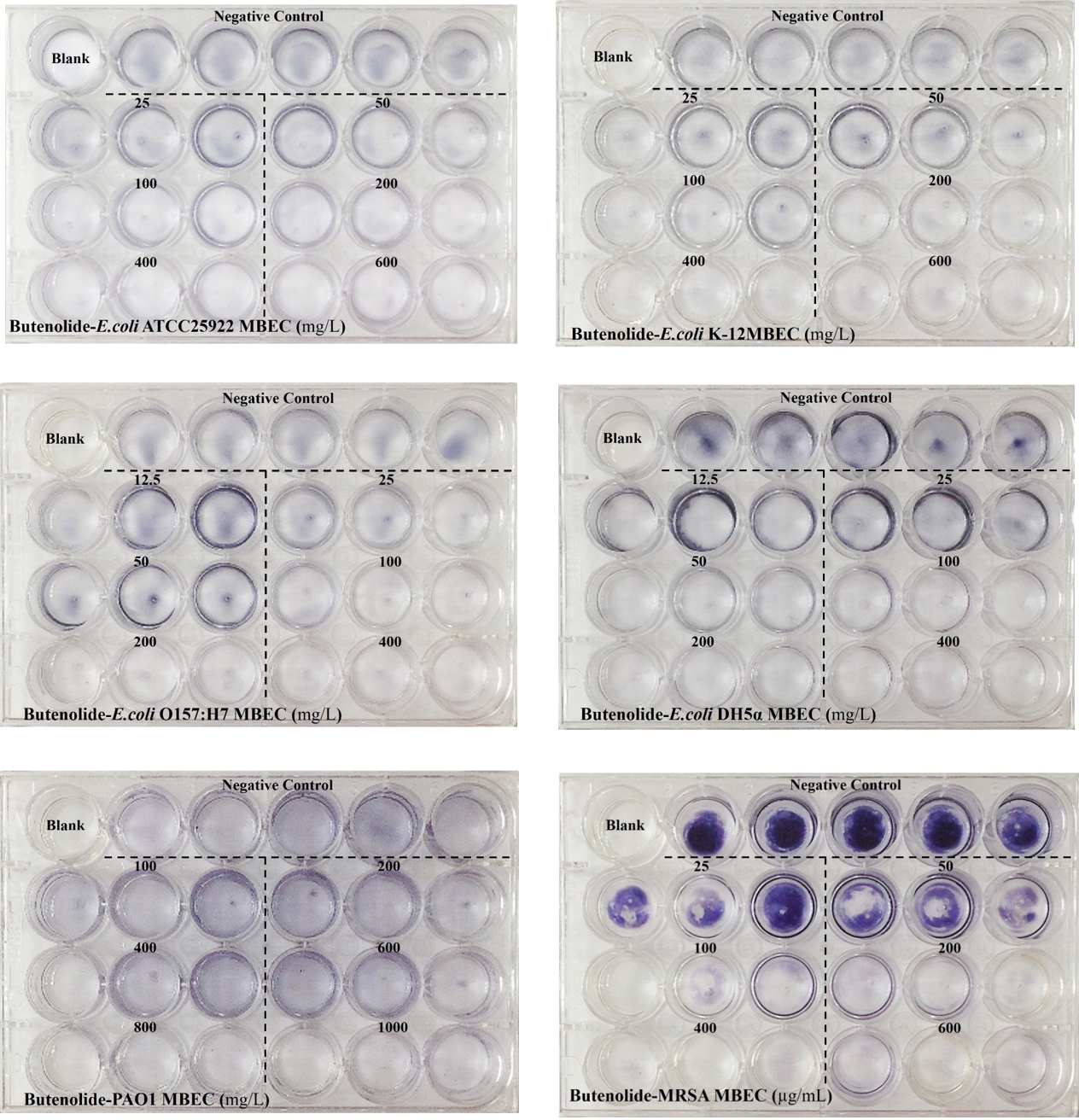


**Fig. S3** Eradication of pre-formed biofilms by various concentraions of butenolide. Six biofilms were allowed to form for 24 h and the viable cells in residual biofilm were measured by MTT staining described in materials and methods. Picture showed colour changes after staining with 0.5% MTT in dark for 2 h. The experiments were performed in triplicates and repeated three times


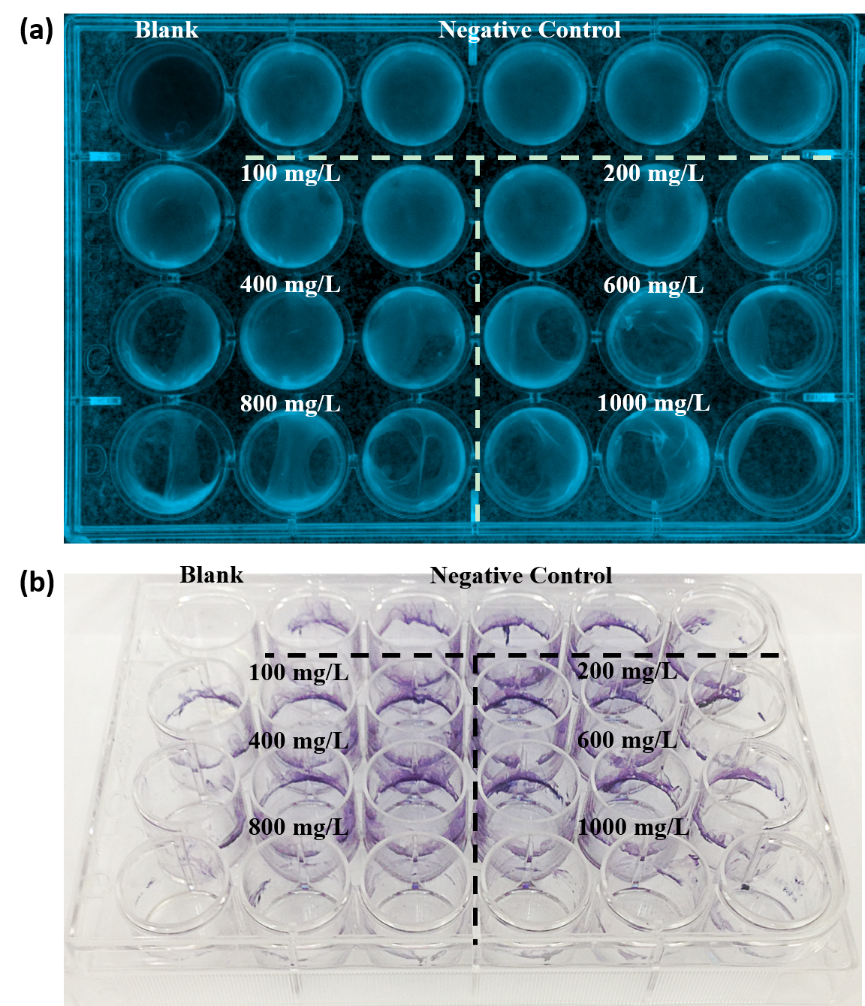


**Fig. S4** Biofilms morphology of PAO1 treated with various concentrations of butenolide (BU). (a) Biofilms were teared following increased BU conctretions, begining at 400 mg/L. (b) Under biofilm superficial tear effect of PAO1, biofilms were easy to remove from wells, which means BU also has the ability to eradicate pre-formed biofilm of PAO1 at low concentration.


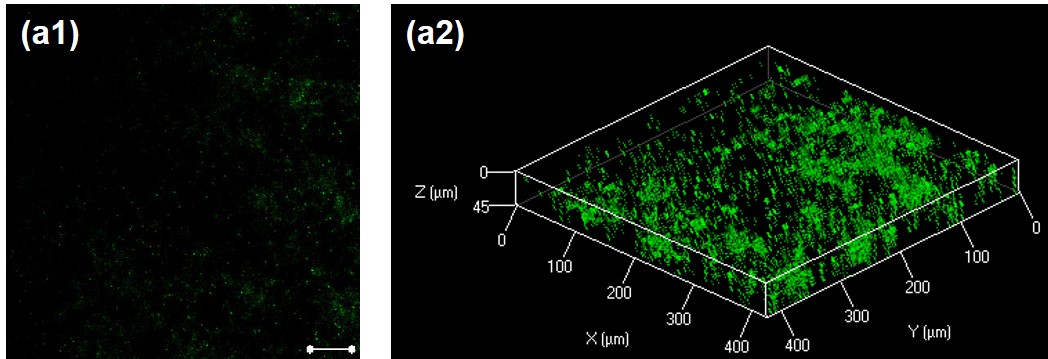


**Fig. S5** CLSM assay of biofilm inhibition of PAO1 exposure to 200 mg/L butenolide (BU). Results of COMSTAT showed biofilm average coverage is 6.63 ± 2.07% and average thichness is 1.18 ± 0.93 μm exposure to 200 mg/L of BU for 24 h, decreasing 68% and 80% compared to negative control. Scale bar is 50 μm.


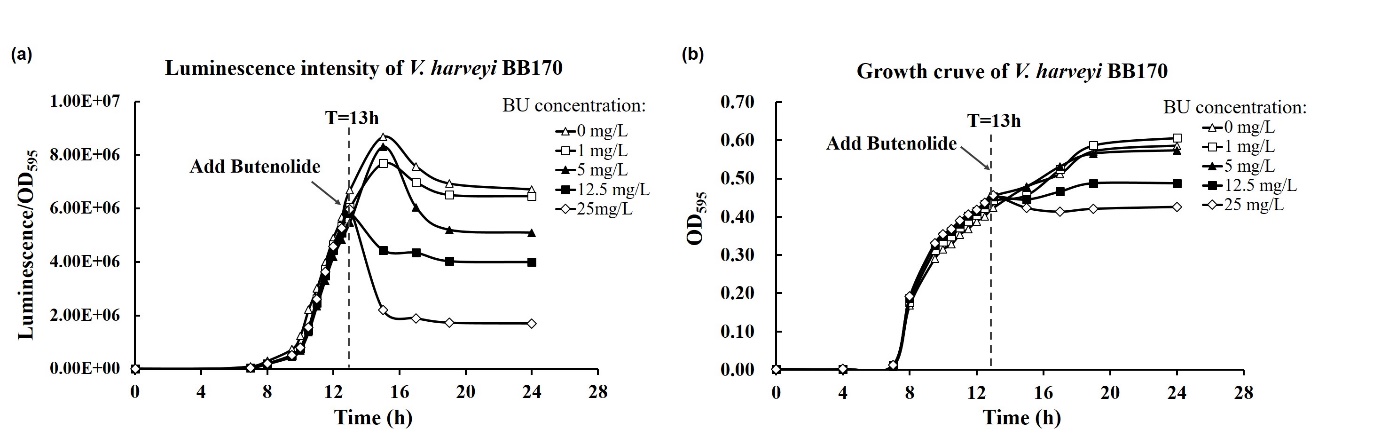


**Fig. S6** The effects of butenolide (BU) on AI-2 mediated QS system were investigated by adding various concentrations of BU after 13 h incubation of reporter strain *Vibrio harveyi* BB170. (a) Luminescence intensity of *V. harveyi* BB170 normalized by total cell density under 1 mg/L, 5 mg/L, 12.5 mg/L and 25 mg/L of BU. (b) Growth curves of *V. harveyi* BB170 under low concentrations of BU (1 mg/L, 5 mg/L, 12.5 mg/L and 25 mg/L). The experiments were performed in triplicates and repeated three times.


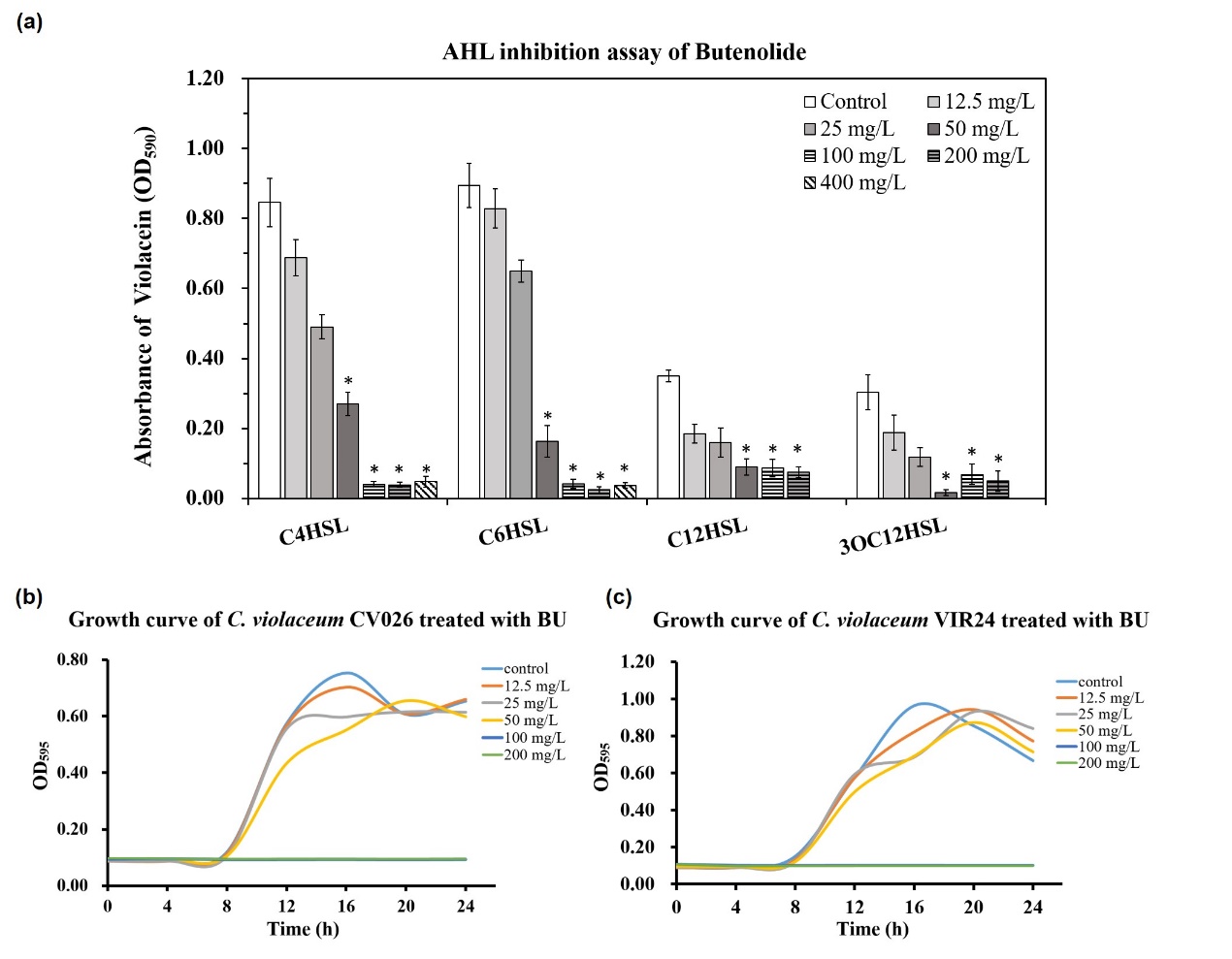


**Fig. S7** The effects of butenolide (BU) on four types AHLs (C4HSL, C6HSL, C12HSL and 3OC12HSL) mediated QS system were investigated according to violacein formation by reporter strains treated with various concentrations of BU. (a) Inhibition of violacein production (four AHL signaling systems) of *C. violaceum* by exposure to various concentrations of BU (12.5 mg/L, 25 mg/L, 50 mg/L, 100 mg/L, 200 mg/L and 400 mg/L). (b) Growth curve of reporter strain *C. violaceum* CV026 treated with a various concentrations of BU. (c) Growth curve of reporter strain *C. violaceum* VIR24 treated with a various concentrations of BU. The experiments were performed in triplicates and repeated three times.


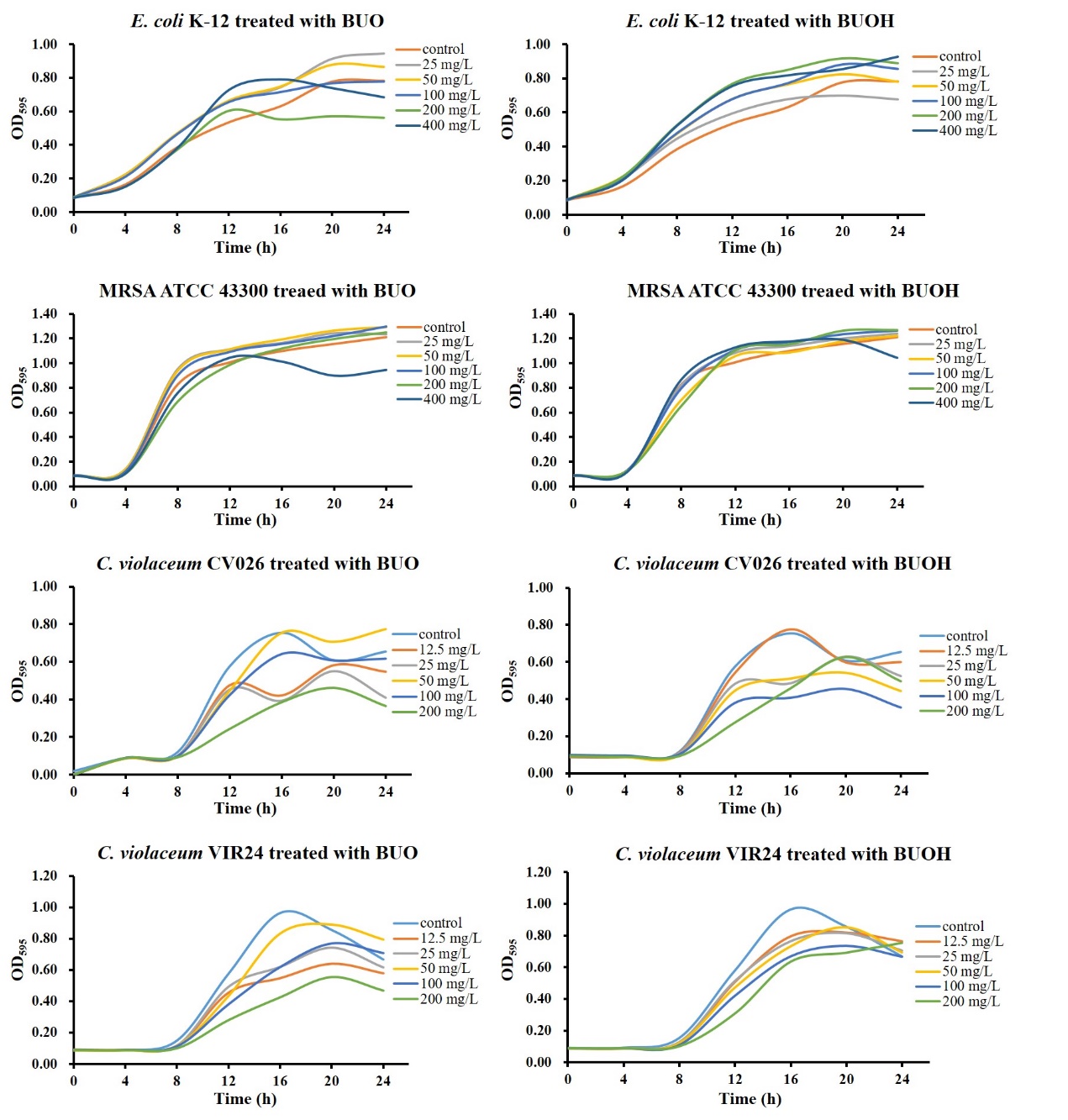


**Fig. S8** Growth curves of biofilm model species *E. Coli* K-12 and MRSA ATCC 43300, treated with various concentrations (12.5 mg/L, 25 mg/L, 50 mg/L, 100 mg/L, 200 mg/L or 400 mg/L) of BUO and BUOH (hydrophilic structural analogs of butenolide). The experiments were performed in triplicates and repeated three times.

**Table S1** Chemical structures and antibiofilm activities of various reported brominate furanones form 2000-2016

| Furanone compounds | Chemical structure | Different names | Biofilm model strains | GRC | MBIC_50_ | MBEC | GRC  /MBIC_50_ | MIC | QS signal inhibition | Ref. |
| --- | --- | --- | --- | --- | --- | --- | --- | --- | --- | --- |
| (5Z)-4-bromo-5-(bromomethylene)-3-butyl-2(5H)-furanone |  | Natural furanone | *Escherichia coli* | 30 mg/L | 60-100 mg/L | – | 0.3-0.5 | – | AI-2 | (Ren et al. 2001; Ren et al. 2004) |
|  |  | furanone | *Bacillus subtilis* | 10 mg/L | >40 mg/L | – | <0.25 | 60 mg/L | AI-2 | (Ren et al. 2002) |
|  |  | Fur-6 | *Salmonella enterica* | – | 100 μM | – | – | – | AHL | (Janssens et al. 2008) |
| (Z)-5-bromomethylene-2(5H)-furanone | 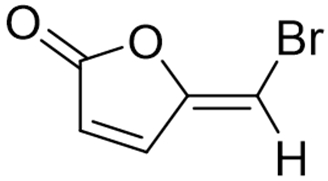 | Fruanone | *Streptococcus anginosus* | <60 μM | >6.0 μM | – | <10 | – | AI-2 | (Lönn-Stensrud et al. 2007) |
|  |  |  | *Streptococcus intermedius* | 60 μM | 6.0 μM | – | 10 | – |  |  |
|  |  |  | *Streptococcus mutans* | 60 μM | >6.0 μM | – | <10 | – |  |  |
|  |  | Furanone 56 | *Pseudomonas aeruginosa* | – | 2.5 μM^a^ | – | – | – | AHL | (Hentzer et al. 2002) |
|  |  | Fur-1 | *Salmonella enterica* | 30 μM | 10-20 μM | – | 1.5-3 | 500 μM | AHL | (Janssens et al. 2008) |
|  |  | F202 | *Staphylococcus epidermidis* | – | 60 μM^b^ | – | – | 1000 μM | AI-2 | (Lönn-Stensrud et al. 2008) |
| (Z-)-4-bromo-5-(bromomethylene)-2(5H)-furanone | 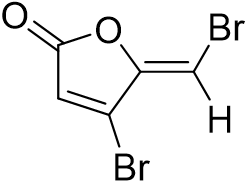 | Fur-3 | *Salmonella enterica* | 40 μM | 7-13 μM | – | 3-5.7 | 500 μM | AHL | (Janssens et al. 2008) |
|  |  | C-30 | *Streptococcus mutans* | 2 mg/L | 2-4 mg/L | – | 1-2 | – | – | (He et al. 2012) |
|  |  | C-30 | *Acidithiobacillus ferrooxidans* | – | 0.5 μM | No^f^ | – | – | – | (Zhao et al. 2015) |
|  |  | BBF^c^ | *Pseudomonas aeruginosa* | – | >10 mg/L^d^ | – | – | – | – | (Wu et al. 2015) |
|  |  |  | *Escherichia coli* | – | >10 mg/L^e^ |  |  |  |  |  |
|  |  |  | *Bacillus subtilis* | – | 10 mg/L |  |  |  |  |  |
| 5-(dibromomethylene)furan-2(5H)-one | 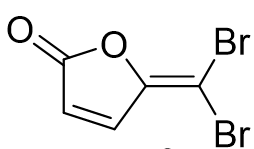 | Fur-2 | *Salmonella enterica* | 40 μM | 11-19 μM | – | 2.1-3.6 | 500 μM | AHL | (Janssens et al. 2008) |
| (Z-)-4-bromo-5-(bromomethylene)-3-ethylfuran-2(5H)-one | 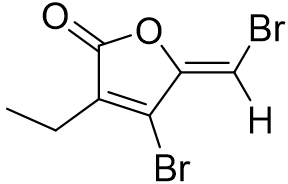 | Fur-5 |  | 200 μM | 45-55 μM | – | 3.5-4.4 | – |  |  |
| (Z)-5-(bromomethylene)-3-hexylfuran-2(5H)-one | 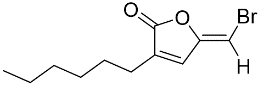 | Fur-8 |  | 150 μM | 45-75 μM | – | 2.0-3.3 | – |  |  |
| (Z)-4-bromo-5-(bromomethylene)-3-methylfuran-2(5H)-one | 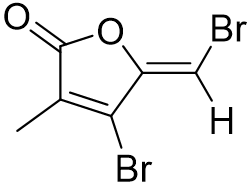 | BF-8 | *Escherichia coli* | >100 mg/L | <60 mg/L^g^ | – | – | – | – | (Han et al. 2008) |
| (Z)-3-bromo-5-(bromomethylene)-furan-2(5H)-one | 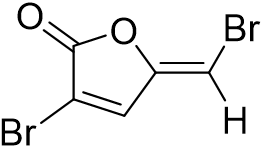 | F206 | *Staphylococcus epidermidis* | – | 60 μM^b^ | – | – | 1600 μM | AI-2 | (Lönn-Stensrud et al. 2008) |

– no detection.

^a^ with additional AHLs.

^b^ coated with compound for 24 h.

^c^ compound added with 1% nafion.

^d^ 23% biofilm inhibition; ^e^ 37% biofilm inhibition; ^g^ 75% biofilm inhibition.

^f^ brominated furanone C-30 had no pre-formed biofilm eradication ability.

**Reference**

Han Y, Hou S, Simon KA, Ren DC, Luk Y-Y (2008) Identifying the important structural elements of brominated furanones for inhibiting biofilm formation by *Escherichia coli*. Bioorg Med Chem Lett 18:1006-1010

Hentzer M, Riedel K, Rasmussen TB, Heydorn A, Andersen JB, Parsek MR, Rice SA, Eberl L, Molin S, Høiby N, Kjelleberg S, Givskov M (2002) Inhibition of quorum sensing in *Pseudomonas aeruginosa* biofilm bacteria by a halogenated furanone compound. Microbiology 148:87-102

He Z, Wang Q, Hu Y, Liang J, Jiang Y, Ma R, Tang Z, Huang Z (2012) Use of the quorum sensing inhibitor furanone C-30 to interfere with biofilm formation by *Streptococcus mutans* and its *luxS* mutant strain. Int J Antimicrob Agents 40:30-35

Janssens JCA, Steenackers H, Robijns S, Gellens E, Levin J, Zhao H, Hermans K, Coster DD, Verhoeven TL, Marchal K (2008) Brominated Furanones Inhibit Biofilm Formation by *Salmonella enterica* Serovar *Typhimurium*. Appl Environ Microbiol 74:6639-6648

Lönn-Stensrud J, Landin MA, Benneche T, Petersen FC, Scheie AA (2008) Furanones, potential agents for preventing *Staphylococcus epidermidis* biofilm infections? J Antimicrob Chemother 63:309-316

Lönn-Stensrud J, Petersen F, Benneche T, Scheie AA (2007) Synthetic bromated furanone inhibits autoinducer-2-mediated communication and biofilm formation in oral streptococci. Mol Oral Microbiol 22:340-346Martinelli D, Grossmann G, Séquin U, Brandl H, Bachofen R (2004) Effects of natural and chemically synthesized furanones on quorum sensing in *Chromobacterium violaceum*. BMC Microbiol 4:25

Ren DC, Sims JJ, Wood TK (2002) Inhibition of biofilm formation and swarming of *Bacillus subtilis* by (*5Z*)-4-bromo-5-(bromomethylene)-3-butyl-2(*5H*)-furanone. Lett Appl Microbiol 34:293-299

Ren DC, Sims JJ, Wood TK (2001) Inhibition of biofilm formation and swarming of *Escherichia coli* by (*5Z*)-4-bromo-5-(bromomethylene)-3-butyl-2(*5H*)-furanone. Environ Microbiol 3:731-736

Ren DC, Bedzyk LA, Ye RW, Thomas SM, Wood TK (2004) Differential gene expression shows natural brominated furanones interfere with the autoinducer-2 bacterial signaling system of *Escherichia coli*. Biotechnol Bioeng 88:630-642

Zhao Y, Chen P, Nan W, Zhi D, Liu R, Li H (2015) The use of (5Z)-4-bromo-5-(bromomethylene)-2(5H)-furanone for controlling acid mine drainage through the inhibition of *Acidithiobacillus ferrooxidans* biofilm formation. Bioresour Technol 186:52-57

Wu Y, Quan X, Si X (2015) Incorporation of brominated furanone into Nafion polymer enhanced anti-biofilm efficacy. Int Biodeterior Biodegradation 99:39-44
